# Effect of Sarcopenia on the Outcomes of Radiofrequency Ablation of Medial Branch Nerves for Lumbar Facet Arthropathy in Patients Aged 60 Years and Older: A Retrospective Analysis

**DOI:** 10.3390/jpm15080344

**Published:** 2025-08-01

**Authors:** Seung Hee Yoo, Won-Joong Kim

**Affiliations:** Department of Anesthesiology and Pain Medicine, College of Medicine, Ewha Womans University, Seoul 07985, Republic of Korea; yoosh0710@naver.com

**Keywords:** radiofrequency ablation, osteoarthritis, spine, sarcopenia, zygapophyseal joint

## Abstract

**Background/Objectives**: Sarcopenia is defined by the progressive loss of muscle mass, strength, and/or physical performance associated with aging. Radiofrequency ablation (RFA) of the medial branch nerves is a well-established and effective treatment for lumbar facetogenic pain. While sarcopenia is associated with poor outcomes following epidural steroid injections and lumbar spine surgeries, its impact on clinical outcomes in patients undergoing RFA for facetogenic pain remains unexplored. This study aims to evaluate the influence of sarcopenia on treatment outcomes in this patient cohort. **Methods**: Patients were classified into sarcopenia (*n* = 35) and non-sarcopenia groups (*n* = 67) based on predefined psoas muscle index (PMI) thresholds. The primary outcomes included changes in back pain intensity and the proportion of responders at 1, 3, and 6 months following RFA. The secondary outcome was to identify demographic, clinical, and sarcopenia-related factors predictive of treatment response at each follow-up interval. **Results**: Both groups demonstrated statistically significant improvements in pain scores compared to baseline at all follow-up points. However, the median pain scores at 3 months post-RFA remained significantly higher in the sarcopenia group. Despite this, the proportion of responders did not differ significantly between the two groups at any time point. At 3 months, the absence of prior spinal surgery was identified as a significant predictor of treatment response. At 6 months, favorable outcomes were significantly associated with the absence of diabetes, no history of spinal surgery, and a higher PMI. **Conclusions**: Sarcopenia may influence the extent of pain improvement following medial branch nerve RFA. Additionally, patient-specific factors, such as diabetes, prior spinal surgery, and PMI, should be considered when predicting treatment outcomes.

## 1. Introduction

Chronic low back pain is a significant healthcare issue that impairs quality of life and functionality and increases disability. Facetogenic pain accounts for approximately 34.1% of all cases [1], with prevalence increasing with age [2]. However, diagnosing lumbar facetogenic pain remains challenging due to its heterogeneous etiology, variable clinical presentation, unclear physical examination findings, inconsistent radiologic evidence, and the need for confirmation through diagnostic medial branch blocks (MBBs) [3,4]. Neither Magnetic Resonance Imaging (MRI) nor Computed Tomography (CT) can reliably diagnose lumbar facet-mediated pathology as the sole source of pain [5].

Currently, lumbar facetogenic pain is diagnosed using diagnostic facet blocks, including intra-articular facet joint injections and MBBs [6]. MBBs are considered more predictive of successful outcomes following lumbar radiofrequency ablation (RFA) than intra-articular facet injections, although both approaches are more effective than sham procedures [7]. Each facet joint is innervated by two medial branches: one from the corresponding spinal level and another from the level above. For example, the L4 medial branch innervates the inferior portion of the L4-L5 facet joint, while the superior portion is innervated by the L3 medial branch [8]. RFA of the medial branch nerves is a well-established treatment with high success rates for managing lumbar facetogenic pain [9], with strong evidence supporting short-term relief, and moderate evidence for long-term pain relief [10].

Sarcopenia, as defined by the European Working Group on Sarcopenia in Older People (EWGSOP), involves the progressive loss of muscle mass, strength, and/or physical performance with age [11]. Its development and progress involve the interplay of multiple complex biological mechanisms. Firstly, aging results in a reduction in both the number and cross-sectional area (CSA) of muscle fibers, leading to overall muscle atrophy. Secondly, imbalances in protein metabolism—specifically, between muscle protein synthesis and degradation—contribute to the progressive decline in muscle mass. Additionally, age-related hormonal alterations, including decreased levels of anabolic hormones such as growth hormone and testosterone, along with elevated concentrations of catabolic agents like cortisol and pro-inflammatory cytokines, further exacerbate muscle deterioration. Finally, changes in gene expression and disruptions in cellular processes such as apoptosis can adversely affect muscle tissue health and play a pivotal role in the pathogenesis of sarcopenia [12].

Various methods exist for assessing appendicular skeletal muscle mass [13], including dual-energy X-ray absorptiometry and bioelectrical impedance analysis. Radiological indices like the psoas muscle index (PMI), psoas-lumbar vertebral index (PLVI), and paraspinal muscle index (PSMI) are frequently used to estimate muscle mass and assess central sarcopenia [14,15,16]. Among these, PMI is highly correlated with traditional sarcopenia assessment tools and is considered a reliable method for evaluating muscle health, particularly in Asian countries such as Japan and South Korea [17,18,19]. Similarly, PLVI has been identified as a valid radiological marker for central sarcopenia and frailty, particularly in patients undergoing spinal surgery [15,20,21]. PSMI has likewise been proposed as an objective metric reflecting paraspinal muscle quality and sarcopenia severity [16].

These imaging parameters offer several advantages for sarcopenia assessment: they provide objective, quantifiable data that are easier to obtain than traditional tools, eliminate the need for physical performance tests, and often use existing cross-sectional imaging studies. Additionally, they do not require patient cooperation or detailed medical histories, making them especially useful for elderly individuals with cognitive impairments or communication challenges.

Recent studies have shown that sarcopenia is associated with adverse postoperative outcomes, increased morbidity, and elevated mortality across a range of medical conditions [12]. While sarcopenia has been linked to poor outcomes following epidural procedures [22,23,24] and lumbar spine surgeries [25], its impact on clinical outcomes in patients with facetogenic pain undergoing RFA has not been specifically studied. Accordingly, the aim of this study is to investigate the effect of sarcopenia on treatment outcomes within this particular patient group.

## 2. Materials and Methods

This study was approved by the Institutional Review Board (IRB) of Ewha Womans University Mokdong Hospital (EUMC 2024-10-001-001), which waived the requirement for informed consent. Electronic medical records of patients who underwent RFA of the lumbar medial branch nerves between September 2019 and November 2023 were retrospectively reviewed for inclusion. All patients underwent two diagnostic MBBs with 0.5 mL of 2% lidocaine at each target level, following negative aspiration for blood [26]. An MBB was considered successful if it produced a >50% reduction in pain intensity. Patients who met this criterion were subsequently evaluated for RFA when clinically appropriate.

The inclusion criteria were (1) age > 60 years, (2) predominantly axial low back pain lasting at least three months, and (3) inadequate response to conservative treatments, including physical therapy, integrative therapies, or pharmacological management.

The exclusion criteria were (1) any epidural injection or other spinal interventions within 1 month prior to RFA, (2) incomplete medical records, (3) absence of lumbar CT imaging, and (4) follow-up duration of <6 months.

### 2.1. Radiofrequency Ablation Procedure

The target point for each MBB was identified at the junction between the superomedial border of the transverse process and the inferolateral neck of the superior articular process (SAP) for L1–L4 levels, and at the groove between the sacral ala and the S1 articular process for the L5 dorsal ramus. An anatomical study [27] showed that the L1–L4 medial branches course posteriorly along the lateral neck of the SAP of the inferior vertebra. Each branch lies superior to the junction of the SAP and transverse process, following the contour of the lateral neck before traveling posteriorly to the mamillo-accessory notch, a concave bony area between the mamillary and accessory processes, which defines the posterior margin of the lateral neck of the SAP. The L5 medial branch follows a similar course along the lateral neck of the SAP but is positioned superior to the junction of the sacral ala and SAP.

RFA of the medial branch nerves was performed under sterile conditions with the patient in a prone position. All procedures were carried out under local anesthesia (1% lidocaine), without sedation. The lumbar spine was prepped using standard sterile technique, and a C-arm fluoroscope was positioned obliquely and caudocephalad to align the cannula with the target nerve trajectory. A 22-gauge curved RF cannula with a 10-mm active tip was inserted to the same target point as the MBB before being advanced under a coaxial view until it contacted the bony junction between the superomedial border of the transverse process and the inferolateral neck of the SAP at levels above L5. For L5 dorsal ramus ablation, the cannula was positioned in the groove between the S1 articular process and sacral ala.

At each level, sensory stimulation (50 Hz) and motor stimulation (2 Hz) confirmed proper electrode placement by eliciting multifidus muscle contraction. Once confirmed, 0.5 mL of 2% lidocaine mixed with 10 mg of triamcinolone was injected to minimize discomfort, enhance lesion size, and reduce post-procedural neuritis [28]. Thermal lesions were created using an RF generator at 80 °C for 90 s, repeated three times.

### 2.2. Clinical Data Collection

Demographic and clinical data included age, sex, height, body mass index (BMI), diabetes mellitus status, history of spinal surgery at the RFA-treated site, presence of compression fractures or spondylolisthesis at the treated site, pain duration prior to RFA, baseline and post-procedural pain scores using the 11-point Numeric Rating Scale (NRS) at 1, 3, and 6 months, number of responders at each time point, treated levels, and RFA laterality. A responder was defined as a patient who experienced a ≥30% reduction in pain score at follow-up without increased analgesic use compared to baseline [29]. Sarcopenia-related parameters included the PMI, PLVI, and PSMI. PMI was calculated by manually outlining the bilateral psoas muscles at the L3 vertebral level, measuring their total cross-sectional area (CSA), and normalizing it to the patient’s height squared (mm^2^/m^2^) [14,30,31]. PLVI was determined by dividing the mean CSA of the bilateral psoas muscles by the CSA of the L4 vertebral body at the inferior endplate level. This was calculated using the following formula: (left psoas CSA + right psoas CSA)/2/L4 vertebral body CSA [20,21]. PSMI was calculated by measuring the CSA of the multifidus and erector spinae muscles at the L3–L4 disc level, then normalizing their sum to height squared (mm^2^/m^2^) [24].

All imaging measurements were manually performed using picture archiving and communication system (PACS) software (7.0.0.8) and independently reviewed by two pain physicians blinded to the patients’ clinical data.

### 2.3. Outcome Measures

Patients were categorized into sarcopenia and non-sarcopenia groups using PMI thresholds at the L3 level: <564.2 mm^2^/m^2^ for men and <414.5 mm^2^/m^2^ for women [14,30]. Primary outcomes included changes in back pain intensity (NRS) and the proportion of responders at 1, 3, and 6 months post-RFA. The secondary outcome was to identify demographic, clinical, and sarcopenia-related predictors of treatment response at these time points.

### 2.4. Statistical Analysis

Statistical analyses were conducted using SPSS version 18.0 (IBM Corp., Chicago, IL, USA). The Shapiro–Wilk test assessed normality of continuous variables. Based on distribution, continuous variables are presented as mean ± standard deviation (SD) or median with interquartile range (IQR), and categorical variables as frequencies and percentages.

Between-group comparisons were performed using the Student’s *t*-test or the Mann-Whitney U test for continuous variables and the chi-squared test for categorical variables. Within-group comparisons were analyzed using the Wilcoxon signed-rank test. A *p*-value < 0.05 was considered statistically significant.

A post hoc power analysis was conducted based on the effect size derived from the Mann-Whitney U test comparing 3-month NRS scores between groups. The effect size (r) was converted to an approximate Cohen’s d, and statistical power was calculated at an alpha level (α) of 0.05.

To identify predictors of RFA response, logistic regression analyses were performed. Variables with *p*-values < 0.10 in univariate analyses were included in the multivariate logistic regression model. A *p*-value < 0.05 was considered significant in the multivariate analysis.

## 3. Results

**Figure 1:**
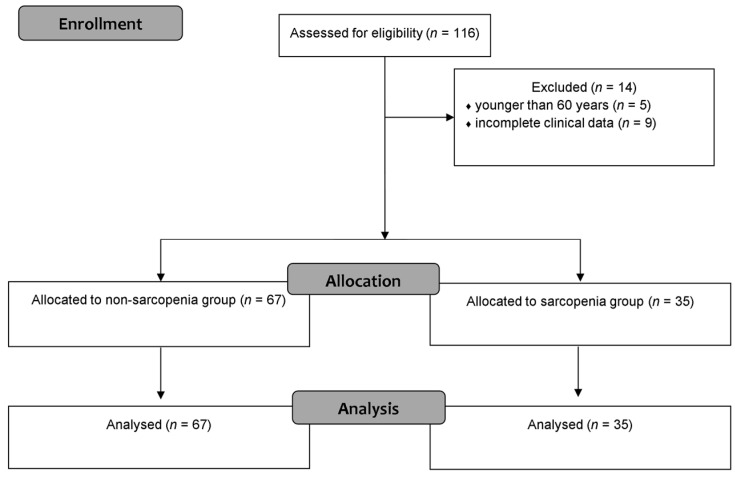
CONSORT flow diagram.

During the study period, 116 patients who underwent RFA were assessed for eligibility. Of these, 14 were excluded based on the study criteria: five were under 60 years of age, and nine had incomplete clinical data. Ultimately, 102 patients were included in the final analysis and categorized into two groups: sarcopenia (*n* = 35) and non-sarcopenia (*n* = 67) (Figure 1).

Table 1 summarizes the baseline demographic and sarcopenia-related characteristics of the study population. The sarcopenia group had significantly lower BMI, lower rates of prior spinal surgery, and a lower prevalence of spondylolisthesis, along with significantly reduced PLVI and PSMI values compared to the non-sarcopenia group.

**Figure 2:**
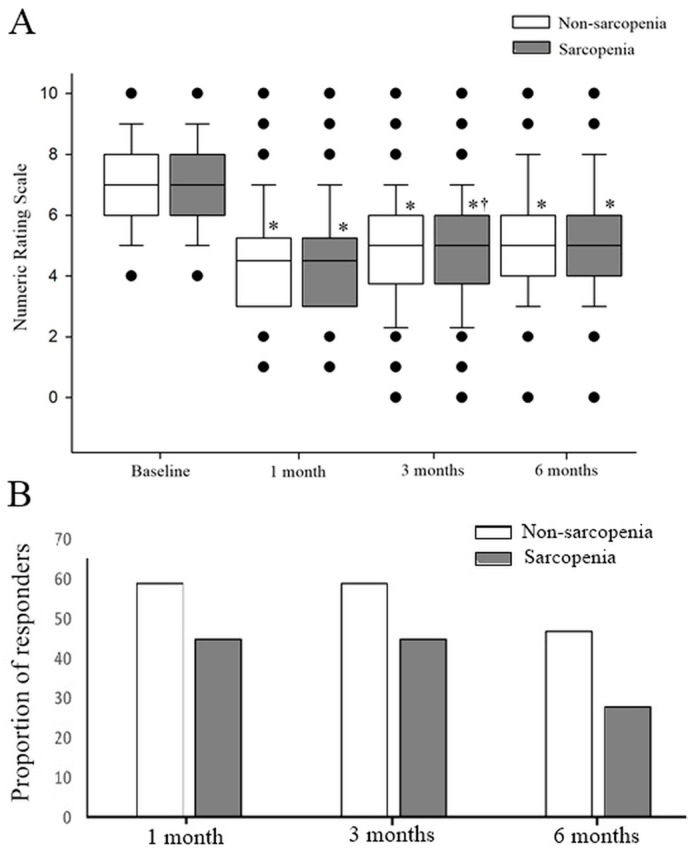
Pain scores and proportion of responders. (**A**) Numeric Rating Scale scores for back pain. (**B**) Proportion of responders. * *p* < 0.05, baseline vs. post-RFAs; Wilcoxon signed-rank test. † *p* < 0.05, non-sarcopenia vs. sarcopenia; Mann-Whitney U test.

Both groups showed statistically significant improvements in pain scores from baseline at all follow-up time points. However, the median pain score remained significantly higher in the sarcopenia group at 3 months post-RFA (Figure 2A). Despite this, the proportion of responders did not significantly differ between groups at any follow-up interval (Figure 2B). For the 3-month NRS comparison, the estimated effect size was r = 0.233, corresponding to a post hoc power of 0.60.

Univariate logistic regression analysis showed that age, sex, height, BMI, compression fractures, spondylolisthesis, PLVI, and PSMI were not significantly associated with treatment response (*p* > 0.10). Table 2 shows the results of the multivariate logistic regression analysis. At 3 months post-RFA, the absence of prior spinal surgery significantly predicted treatment response. At 6 months post-RFA, favorable treatment response was associated with the absence of diabetes, no history of spinal surgery, and higher PMI.

No serious adverse events or complications related to RFA were documented in the patients’ medical records.

## 4. Discussion

In this study, we evaluated the effect of sarcopenia on clinical outcomes following RFA for chronic lumbar facetogenic pain. Although both sarcopenic and non-sarcopenic patients experienced significant pain reduction, the sarcopenia group demonstrated higher median pain scores at 3 months post-procedure. Multivariate analysis identified the absence of prior spinal surgery as a significant predictor of treatment response at 3 months. At 6 months, the absence of diabetes, no history of spinal surgery, and a higher PMI were significant predictors of a favorable outcome.

Several previous studies have investigated the relationship between sarcopenia and outcomes of non-surgical spinal interventions in patients with lumbar spinal disease. For example, in a study examining the effects of percutaneous epidural balloon neuroplasty—using the same PMI threshold as our study—no significant differences in median NRS scores for back and leg pain were observed between patients with and without sarcopenia. However, both groups showed significant improvement at 1, 3, and 6 months compared to baseline [30]. This finding contrasts with our results, which demonstrated a diminished analgesic effect of RFA in the sarcopenia group, particularly in those with low PMI at 3 months post-procedure.

Additionally, two studies assessed the analgesic efficacy of epidural steroid injections in elderly patients with symptomatic degenerative lumbar spinal disease by categorizing patients into good and poor analgesia groups. One study found that pre-procedural handgrip strength was significantly higher in the good analgesia group, whereas PMI did not differ between the two groups [23]. Another study examining paraspinal muscle degeneration reported that fat infiltration grade was significantly higher in the poor analgesia group, while CSA did not differ [24]. Similarly, a study of patients undergoing epidural adhesiolysis identified higher paraspinal muscle fat infiltration as an independent factor associated with poorer outcomes in patients aged ≥65 years. However, CSA was not significantly associated with pain relief [32]. These findings are partially consistent with ours, as PMI predicted treatment response to RFA, whereas PSMI did not.

Although previous studies suggest fat infiltration may affect analgesic outcomes, we did not include fat infiltration as a sarcopenia marker for several reasons. Many studies have failed to show a clear association between pain and facet arthropathy severity on CT or MRI [33]. While CT or MRI was routinely performed pre-RFA to rule out other etiologies, MRI is generally more expensive than CT in Korea. Furthermore, a study by Berg et al. [34] showed that CT provided superior reliability in assessing the overall grade of facet arthropathy, including osteophyte formation and hypertrophy. Consequently, MRIs are often not performed prior to RFA in clinical practice.

As discussed, existing studies on non-surgical spinal interventions have produced mixed results. One reason may be the focus on epidural procedures, with few studies specifically addressing facet-mediated pain. This distinction is clinically relevant, as pain mechanisms and therapeutic responses in spinal disorders are highly heterogeneous. Our findings highlight the limitations of generalized treatment approaches and support mechanism-based, individualized therapeutic strategies in spinal care. Future prospective studies are needed to develop comprehensive, personalized predictive models that integrate these variables and improve clinical decision-making and patient outcomes.

The psoas muscles are critical for spinal biomechanics and maintaining lumbar stability [35]. Our findings suggest that medial branch RFA is less effective in patients with low PMI, and that PMI predicts treatment success at 6 months. Although PMI has not been previously studied as a prognostic factor in facetogenic pain, research supports its prognostic value in spinal surgery. In one study on Asian patients, those with sarcopenia and low PMI experienced longer hospital stays despite similar rates of postoperative complications [36]. In another study of patients aged > 65, those with low PMI required more transfusions and had longer ICU stays after thoracolumbar surgery [37]. Bourassa-Moreau et al. [38] reported that lower psoas CSA was associated with postoperative adverse events and increased 3-month mortality following surgery for metastatic spine disease. Similarly, Gakhar et al. [39] found that reduced psoas area predicted 1-year mortality following spinal decompression.

Our findings indicate no association between PLVI and treatment response. Although PLVI is used to predict prognosis in surgical and interventional settings, study results remain inconsistent. For example, patients with low PLVI undergoing single-level lumbar fusion report worse pain and functional outcomes [40], while those with osteoporotic vertebral compression fractures experience poorer outcomes in terms of postoperative pain, functional recovery, and higher refracture rates [41]. In contrast, low PLVI was not associated with increased infection rates [42] or proximal junctional disease in patients undergoing lumbar arthrodesis [43].

Sarcopenia significantly lowers the multifidus and erector spinae muscle index [44], while paraspinal muscle degeneration strongly correlates with facet joint osteoarthritis [45]. Consistent with this, patients with sarcopenia in our study had lower PSMI. However, PSMI did not correlate with treatment response. Similar studies suggest PMI is a more reliable predictor than paraspinal CSA, which has not been associated with outcomes after lumbar fusion [46,47]. Furthermore, sarcopenia defined by paraspinal muscle CSA has not been found to influence the clinical success of lumbar fusion in patients with degenerative spondylolisthesis [47]. This may be due to the fact that, with aging and degenerative changes, muscle tissue is often replaced by fat, while the overall CSA can remain relatively preserved, thereby diminishing its prognostic value [48].

In addition to sarcopenia-related variables, our findings indicate that the absence of diabetes and prior spinal surgery were associated with better outcomes. A study examining the effects of RFA on the medial branch nerves in elderly patients [8] found that failed back surgery syndrome was an independent predictor of poor outcomes. Spinal fusion sometimes serves as a treatment option for facetogenic pain, with many surgeons intentionally or inadvertently performing medial branch rhizotomies during pedicle screw placement [49]. While data are limited, one retrospective cohort study suggests that diabetic patients with poor glycemic control derive less pain relief from epidural steroid injections [50]. Furthermore, our multivariate logistic regression analysis revealed that pain duration did not significantly predict responder status, which aligns with previous studies [51].

Demographic differences between groups included BMI, prior spinal surgery, and spondylolisthesis. Although the causes are unclear, lower BMI is commonly seen in sarcopenia due to muscle loss, despite possible increases in fat mass [52]. Moreover, a systematic review and meta-analysis also reported that sarcopenia is associated with poorer postoperative quality of life in elderly patients with lumbar degenerative disease [53], which may lead surgeons to recommend conservative care. Additionally, reduced physical activity in sarcopenia may lower the risk of spondylolisthesis, which results from chronic axial loading [54].

This study has several limitations. First, objective assessments of physical functions, such as gait speed and muscle strength, were not included in our evaluation of sarcopenia. Second, the single-center design and predominantly South Korean population may limit the generalizability of the findings. Third, patients were categorized into groups based solely on PMI values, despite there currently being no universally accepted PMI cut-off. Fourth, treatment response was evaluated exclusively by changes in NRS scores, without accounting for other important clinical outcomes such as physical function or patient satisfaction. Finally, although median NRS scores were significantly higher in the sarcopenia group at 3 months, the mean difference did not exceed the minimal clinically important difference of 2 points [29], post hoc power was limited. This raises the possibility of a type II error and warrants cautious interpretation.

## 5. Conclusions

Sarcopenia may reduce the effectiveness of medial branch RFA by compromising psoas and paraspinal muscle integrity, which is essential for spinal stability and load distribution [35]. Reduced muscle mass and quality may increase biomechanical stress on facet joints and adjacent structures, potentially diminishing RFA’s therapeutic efficacy. Patient-specific factors such as diabetes, history of spinal surgery, and PMI should also be considered when predicting treatment outcomes. These findings support a personalized, mechanism-based approach to managing lumbar facet arthropathy.

## Figures and Tables

**Table 1:** Demographic characteristics.

	Non-Sarcopenia (*n* = 67)	Sarcopenia (*n* = 35)	*p*-Value
Age (years)	77.00 [11.00]	73.00 [10.00]	0.086
Sex (M/F)	19 (28.4)/48 (71.6)	7 (20.0)/28 (80.0)	0.358
Height (cm)	155.00 [11.00]	155.00 [8.00]	0.748
BMI (kg/m^2^)	25.00 [4.00]	22.00 [4.00]	0.000
Diabetes mellitus	17 (25.4)	4 (11.4)	0.098
Surgery history	15 (22.4)	2 (5.7)	0.032
Compression fracture	11 (16.4)	6 (17.1)	0.926
Spondylolisthesis	32 (47.8)	9 (25.7)	0.031
Duration (months)	8.00 [19.00]	14.00 [30.00]	0.152
Number of levels treated (2/3/4)	28 (42)/36 (54)/3 (4)	15 (43)/18 (51)/2 (6)	0.951
Laterality (Left/Right)	39 (58.2)/28 (41.8)	17 (48.6)/18 (54.4)	0.353
PLVI	0.597 ± 0.179	0.470 ± 0.154	0.001
PSMI (mm^2^/m^2^)			
Ipsilateral	2780.02 [1014.80]	2388.90 [727.40]	0.000
Contralateral	2878.90 [823.10]	2461.08 [580.41]	0.000

Data are presented as the mean ± standard deviation, median [interquartile range], or number of patients (%). BMI, Body mass index; PLVI, psoas-lumbar vertebral index; PSMI, Paraspinal muscles index.

**Table 2:** Factors associated with treatment response based on multivariate logistic regression analysis.

Variable	Reference Group	OR	95% CI	*p*-Value
3 months after RFA				
Duration	-	0.982	0.961–1.004	0.110
Surgery history	No surgery history	0.246	0.065–0933	0.039
6 months after RFA				
Diabetes mellitus	No diabetes mellitus	0.298	0.104–0.853	0.024
Surgery history	No surgery history	0.165	0.046–0.589	0.006
PMI	-	1.003	1.000–1.006	0.042

CI, confidence interval; OR, odds ratio; PMI, psoas muscle index; RFA, Radiofrequency ablation.

## Data Availability

The data that support the findings of this study are available from the corresponding author upon reasonable request.

## Abbreviations

The following abbreviations are used in this manuscript:

MBB	medial branch block
RFA	Radiofrequency ablation
PMI	psoas muscle index
PLVI	psoas-lumbar vertebral index
PSMI	paraspinal muscle index
CT	computed tomography
MRI	Magnetic Resonance Imaging
BMI	body mass index
NRS	Numeric Rating Scale
CSA	cross-sectional area

## References

[1] Manchikanti L., Kosanovic R., Pampati V., Cash K.A., Soin A., Kaye A.D., Hirsch J.A. (2020). Low Back Pain and Diagnostic Lumbar Facet Joint Nerve Blocks: Assessment of Prevalence, False-Positive Rates, and a Philosophical Paradigm Shift from an Acute to a Chronic Pain Model. Pain Physician.

[2] Laplante B.L., Ketchum J.M., Saullo T.R., DePalma M.J. (2012). Multivariable analysis of the relationship between pain referral patterns and the source of chronic low back pain. Pain Physician.

[3] Manchikanti L., Pampati V., Soin A., Vanaparthy R., Sanapati M.R., Kaye A.D., Hirsch J.A. (2020). Trends of Expenditures and Utilization of Facet Joint Interventions in Fee-For-Service (FFS) Medicare Population from 2009–2018. Pain Physician.

[4] Hao D., Yong R.J., Cohen S.P., Stojanovic M.P. (2023). Medial Branch Blocks and Radiofrequency Ablation for Low Back Pain from Facet Joints. N. Engl. J. Med..

[5] Cohen S.P., Doshi T.L., Kurihara C., Dolomisiewicz E., Liu R.C., Dawson T.C., Hager N., Durbhakula S., Verdun A.V., Hodgson J.A. (2021). Waddell (Nonorganic) Signs and Their Association with Interventional Treatment Outcomes for Low Back Pain. Anesth. Analg..

[6] Cohen S.P., Hurley R.W., Christo P.J., Winkley J., Mohiuddin M.M., Stojanovic M.P. (2007). Clinical predictors of success and failure for lumbar facet radiofrequency denervation. Clin. J. Pain..

[7] Lee D.W., Pritzlaff S., Jung M.J., Ghosh P., Hagedorn J.M., Tate J., Scarfo K., Strand N., Chakravarthy K., Sayed D. (2021). Latest Evidence-Based Application for Radiofrequency Neurotomy (LEARN): Best Practice Guidelines from the American Society of Pain and Neuroscience (ASPN). J. Pain. Res..

[8] Chen Y.S., Liu B., Gu F., Sima L. (2022). Radiofrequency Denervation on Lumbar Facet Joint Pain in the Elderly: A Randomized Controlled Prospective Trial. Pain Physician.

[9] Falco F.J., Manchikanti L., Datta S., Sehgal N., Geffert S., Onyewu O., Zhu J., Coubarous S., Hameed M., Ward S.P. (2012). An update of the effectiveness of therapeutic lumbar facet joint interventions. Pain Physician.

[10] Boswell M.V., Colson J.D., Sehgal N., Dunbar E.E., Epter R. (2007). A systematic review of therapeutic facet joint interventions in chronic spinal pain. Pain Physician.

[11] Cruz-Jentoft A.J., Bahat G., Bauer J., Boirie Y., Bruyere O., Cederholm T., Cooper C., Landi F., Rolland Y., Sayer A.A. (2019). Sarcopenia: Revised European consensus on definition and diagnosis. Age Ageing.

[12] Bentov I., Kaplan S.J., Pham T.N., Reed M.J. (2019). Frailty assessment: From clinical to radiological tools. Br. J. Anaesth..

[13] Cruz-Jentoft A.J., Baeyens J.P., Bauer J.M., Boirie Y., Cederholm T., Landi F., Martin F.C., Michel J.P., Rolland Y., Schneider S.M. (2010). Sarcopenia: European consensus on definition and diagnosis: Report of the European Working Group on Sarcopenia in Older People. Age Ageing.

[14] Amini N., Spolverato G., Gupta R., Margonis G.A., Kim Y., Wagner D., Rezaee N., Weiss M.J., Wolfgang C.L., Makary M.M. (2015). Impact Total Psoas Volume on Short- and Long-Term Outcomes in Patients Undergoing Curative Resection for Pancreatic Adenocarcinoma: A New Tool to Assess Sarcopenia. J. Gastrointest. Surg..

[15] Ebbeling L., Grabo D.J., Shashaty M., Dua R., Sonnad S.S., Sims C.A., Pascual J.L., Schwab C.W., Holena D.N. (2014). Psoas:lumbar vertebra index: Central sarcopenia independently predicts morbidity in elderly trauma patients. Eur. J. Trauma. Emerg. Surg..

[16] Pinter Z.W., Salmons H.I.t., Townsley S., Omar A., Freedman B.A., Currier B.L., Elder B.D., Nassr A.N., Bydon M., Wagner S.C. (2022). Multifidus Sarcopenia Is Associated with Worse Patient-reported Outcomes Following Posterior Cervical Decompression and Fusion. Spine.

[17] Shimizu T., Miyake M., Hori S., Ichikawa K., Omori C., Iemura Y., Owari T., Itami Y., Nakai Y., Anai S. (2020). Clinical Impact of Sarcopenia and Inflammatory/Nutritional Markers in Patients with Unresectable Metastatic Urothelial Carcinoma Treated with Pembrolizumab. Diagnostics.

[18] Tsukagoshi M., Yokobori T., Yajima T., Maeno T., Shimizu K., Mogi A., Araki K., Harimoto N., Shirabe K., Kaira K. (2020). Skeletal muscle mass predicts the outcome of nivolumab treatment for non-small cell lung cancer. Medicine.

[19] Kim J.S., Kim W.Y., Park H.K., Kim M.C., Jung W., Ko B.S. (2017). Simple Age Specific Cutoff Value for Sarcopenia Evaluated by Computed Tomography. Ann. Nutr. Metab..

[20] Sim J.H., Lee S.H., Kim J.W., Koh W.U., Kim H.T., Ro Y.J., Kim H.J. (2021). Low Psoas Lumbar Vertebral Index Is Associated with Mortality after Hip Fracture Surgery in Elderly Patients: A Retrospective Analysis. J. Pers. Med..

[21] An S.M., Chae J.S., Lee H.J., Cho S., Im J. (2024). Association of Psoas: Lumbar Vertebral Index (PLVI) with Postherpetic Neuralgia in Patients Aged 60 and Older with Herpes Zoster. J. Clin. Med..

[22] Kwon H.J., Kim C.S., Kim S., Yoon S.H., Koh J., Kim Y.K., Choi S.S., Shin J.W., Kim D.H. (2023). Association between fatty infiltration in the cervical multifidus and treatment response following cervical interlaminar epidural steroid injection. Korean J. Pain..

[23] Kim S.H., Park S.J., Yoon K.B., Jun E.K., Cho J., Kim H.J. (2022). Influence of Handgrip Strength and Psoas Muscle Index on Analgesic Efficacy of Epidural Steroid Injection in Patients with Degenerative Lumbar Spinal Disease. Pain Physician.

[24] Kim H.J., Rho M., Yoon K.B., Jo M., Lee D.W., Kim S.H. (2022). Influence of cross-sectional area and fat infiltration of paraspinal muscles on analgesic efficacy of epidural steroid injection in elderly patients. Pain. Pr..

[25] Inose H., Yamada T., Hirai T., Yoshii T., Abe Y., Okawa A. (2018). The impact of sarcopenia on the results of lumbar spinal surgery. Osteoporos. Sarcopenia.

[26] Van Zundert J., Mekhail N., Vanelderen P., van Kleef M. (2010). Diagnostic medial branch blocks before lumbar radiofrequency zygapophysial (facet) joint denervation: Benefit or burden?. Anesthesiology.

[27] Tran J., Peng P., Loh E. (2022). Anatomical study of the medial branches of the lumbar dorsal rami: Implications for image-guided intervention. Reg. Anesth. Pain. Med..

[28] Dobrogowski J., Wrzosek A., Wordliczek J. (2005). Radiofrequency denervation with or without addition of pentoxifylline or methylprednisolone for chronic lumbar zygapophysial joint pain. Pharmacol. Rep..

[29] Dworkin R.H., Turk D.C., Wyrwich K.W., Beaton D., Cleeland C.S., Farrar J.T., Haythornthwaite J.A., Jensen M.P., Kerns R.D., Ader D.N. (2008). Interpreting the clinical importance of treatment outcomes in chronic pain clinical trials: IMMPACT recommendations. J. Pain..

[30] Han Y.A., Kwon H.J., Lee K., Son M.G., Kim H., Choi S.S., Shin J.W., Kim D.H. (2023). Impact of Sarcopenia on Percutaneous Epidural Balloon Neuroplasty in Patients with Lumbar Spinal Stenosis: A Retrospective Analysis. Medicina.

[31] Zager Y., Khalilieh S., Ganaiem O., Gorgov E., Horesh N., Anteby R., Kopylov U., Jacoby H., Dreznik Y., Dori A. (2021). Low psoas muscle area is associated with postoperative complications in Crohn’s disease. Int. J. Color. Dis..

[32] Kang M., Kim S.H., Jo M., Jung H.E., Bae J., Kim H.J. (2023). Evaluation of Paraspinal Muscle Degeneration on Pain Relief after Percutaneous Epidural Adhesiolysis in Patients with Degenerative Lumbar Spinal Disease. Medicina.

[33] Gellhorn A.C., Katz J.N., Suri P. (2013). Osteoarthritis of the spine: The facet joints. Nat. Rev. Rheumatol..

[34] Berg L., Thoresen H., Neckelmann G., Furunes H., Hellum C., Espeland A. (2019). Facet arthropathy evaluation: CT or MRI?. Eur. Radiol..

[35] Kim J.B., Park S.W., Lee Y.S., Nam T.K., Park Y.S., Kim Y.B. (2015). The Effects of Spinopelvic Parameters and Paraspinal Muscle Degeneration on S1 Screw Loosening. J. Korean Neurosurg. Soc..

[36] Kumar A.A., Wong W.S., Zheng Y., Leow B.H.W., Low Y.L., Tan L.F., Teo K., Nga V.D.W., Yeo T.T., Lim M.J.R. (2024). Effect of psoas muscle index on early postoperative outcomes in surgically treated spinal tumours in an Asian population. J. Clin. Neurosci..

[37] Pernik M.N., Hicks W.H., Akbik O.S., Nguyen M.L., Luu I., Traylor J.I., Deme P.R., Dosselman L.J., Hall K., Wingfield S.A. (2023). Psoas Muscle Index as a Predictor of Perioperative Outcomes in Geriatric Patients Undergoing Spine Surgery. Glob. Spine J..

[38] Bourassa-Moreau E., Versteeg A., Moskven E., Charest-Morin R., Flexman A., Ailon T., Dalkilic T., Fisher C., Dea N., Boyd M. (2020). Sarcopenia, but not frailty, predicts early mortality and adverse events after emergent surgery for metastatic disease of the spine. Spine J..

[39] Gakhar H., Dhillon A., Blackwell J., Hussain K., Bommireddy R., Klezl Z., Williams J. (2015). Study investigating the role of skeletal muscle mass estimation in metastatic spinal cord compression. Eur. Spine J..

[40] Sun K., Zhu H., Huang B., Li J., Liu G., Jiao G., Chen G. (2024). MRI-based central sarcopenia negatively impacts the therapeutic effectiveness of single-segment lumbar fusion surgery in the elderly. Sci. Rep..

[41] Sun K., Liu J., Zhu H., Wang J., Wan H., Huang B., Zhang Q., Chen G. (2024). Lower psoas mass indicates worse prognosis in percutaneous vertebroplasty-treated osteoporotic vertebral compression fracture. Sci. Rep..

[42] Ruffilli A., Manzetti M., Cerasoli T., Barile F., Viroli G., Traversari M., Salamanna F., Fini M., Faldini C. (2022). Osteopenia and Sarcopenia as Potential Risk Factors for Surgical Site Infection after Posterior Lumbar Fusion: A Retrospective Study. Microorganisms.

[43] Ruffilli A., Manzetti M., Barile F., Ialuna M., Cerasoli T., Viroli G., Salamanna F., Contartese D., Giavaresi G., Faldini C. (2023). Complications after Posterior Lumbar Fusion for Degenerative Disc Disease: Sarcopenia and Osteopenia as Independent Risk Factors for Infection and Proximal Junctional Disease. J. Clin. Med..

[44] Fang T., Xue Z., Zhou Q., Gao J., Mi J., Yang H., Zhou F., Liu H., Zhang J. (2025). Impact of Paraspinal Sarcopenia on Clinical Outcomes in Intervertebral Disc Degeneration Patients Following Percutaneous Transforaminal Endoscopic Lumbar Discectomy. Orthop. Surg..

[45] Yu B., Jiang K., Li X., Zhang J., Liu Z. (2017). Correlation of the Features of the Lumbar Multifidus Muscle with Facet Joint Osteoarthritis. Orthopedics.

[46] Akbik O.S., Al-Adli N., Pernik M.N., Hicks W.H., Hall K., Aoun S.G., Bagley C.A. (2023). A Comparative Analysis of Frailty, Disability, and Sarcopenia with Patient Characteristics and Outcomes in Adult Spinal Deformity Surgery. Glob. Spine J..

[47] McKenzie J.C., Wagner S.C., Sebastian A., Casper D.S., Mangan J., Stull J., Hilibrand A.S., Vaccaro A.R., Kepler C. (2019). Sarcopenia does not affect clinical outcomes following lumbar fusion. J. Clin. Neurosci..

[48] Teichtahl A.J., Urquhart D.M., Wang Y., Wluka A.E., Wijethilake P., O’Sullivan R., Cicuttini F.M. (2015). Fat infiltration of paraspinal muscles is associated with low back pain, disability, and structural abnormalities in community-based adults. Spine J..

[49] Markwalder T.M., Merat M. (1994). The lumbar and lumbosacral facet-syndrome. Diagnostic measures, surgical treatment and results in 119 patients. Acta Neurochir..

[50] Wong F., Namdari B., Dupler S., Kovac M.F., Makarova N., Dalton J.E., Turan A. (2016). No difference in pain reduction after epidural steroid injections in diabetic versus nondiabetic patients: A retrospective cohort study. J. Anaesthesiol. Clin. Pharmacol..

[51] Streitberger K., Muller T., Eichenberger U., Trelle S., Curatolo M. (2011). Factors determining the success of radiofrequency denervation in lumbar facet joint pain: A prospective study. Eur. Spine J..

[52] Romero-Corral A., Montori V.M., Somers V.K., Korinek J., Thomas R.J., Allison T.G., Mookadam F., Lopez-Jimenez F. (2006). Association of bodyweight with total mortality and with cardiovascular events in coronary artery disease: A systematic review of cohort studies. Lancet.

[53] Wu W.T., Lee T.M., Han D.S., Chang K.V. (2021). The Prevalence of Sarcopenia and Its Impact on Clinical Outcomes in Lumbar Degenerative Spine Disease-A Systematic Review and Meta-Analysis. J. Clin. Med..

[54] Kalichman L., Hunter D.J. (2008). Diagnosis and conservative management of degenerative lumbar spondylolisthesis. Eur. Spine J..

